# Glucose-potassium ratio: a prognostic biomarker enhancing outcome prediction in mild-to-moderate traumatic brain injury

**DOI:** 10.3389/fneur.2025.1577390

**Published:** 2025-05-30

**Authors:** Jiayin Wang, Chaocan Hong, Qiangqiang Feng, Baofang Wu, Shaojie Li, Changhu Yan, Hongzhi Gao

**Affiliations:** ^1^Department of Neurosurgery, The Second Affiliated Hospital of Fujian Medical University, Quanzhou, China; ^2^Department of Neurosurgery, Jinjiang Hospital of Traditional Chinese Medicine, Jinjiang, China

**Keywords:** serum glucose and potassium ratio, mild-to-moderate traumatic brain injury, prognosis, restricted cubic spline, propensity score matching, receiver operating characteristic

## Abstract

**Background:**

To determine the prognostic value of the serum glucose-to-potassium ratio (GPR) in patients with mild-to-moderate traumatic brain injury (MM-TBI).

**Methods:**

Clinical data from patients admitted to the Second Affiliated Hospital of Fujian Medical University with MM-TBI between January 1, 2018 and December 31, 2022 were analyzed. Univariate logistic regression was performed to identify potential risk factors for poor prognosis, followed by multivariate logistic regression to evaluate the Rotterdam score and GPR as independent prognostic factors. The stability of the relationship between GPR and prognosis was confirmed using trend tests, multiple regression models, and restricted cubic splines (RCS). Propensity score matching (PSM) was used to adjust for selection bias, receiver operating characteristic (ROC) curves were used to evaluate predictive accuracy, and Kaplan–Meier (K-M) curves were used to assess 90-day prognosis.

**Results:**

GPR (*p* = 0.0021) and Rotterdam score (*p* < 0.0001) were found to be independent prognostic factors. Trend tests and RCS analyses confirmed a stable, nonlinear relationship between GPR and prognosis. The area under the ROC curve was 0.670 for GPR alone, which increased to 0.850 when combined with the Rotterdam score. PSM analysis revealed a significant difference in GPR between the favorable and unfavorable prognosis groups (*p* = 0.004). K–M curves showed that GPR was strongly predictive of 90-day outcomes (*p* < 0.0001).

**Conclusion:**

GPR is a reliable and easily accessible prognostic indicator for MM-TBI. Moreover, its prognostic value is enhanced when GPR is combined with the Rotterdam score.

## Introduction

Traumatic brain injury (TBI), the leading cause of mortality and disability among young adults, is a substantial medical and social burden worldwide (1). An estimated 50–60 million new cases of TBI occur annually with a global economic burden of approximately $400 billion. TBI is typically classified as mild, moderate, or severe based on the Glasgow Coma Scale (GCS) score at the time of admission, which reflects the extent of the patient’s neurological impairment (2). Patients with mild-to-moderate traumatic brain injury (MM-TBI), accounting for approximately 90% of all TBIs, often present with subtle clinical symptoms and focal neurological deficits. Among this group, 10%–20% of patients experience further neurological deterioration, with 7% requiring subsequent surgical intervention (3).

Many individuals with MM-TBI do not seek timely medical care or they are managed by non-specialized healthcare providers, which often leads to an underestimation of the true incidence and prevalence of MM-TBI (4). Therefore, the early and accurate risk assessment of MM-TBI is crucial to prevent secondary injury progression and minimize the risk of death and/or long-term disability. Conventional prognostic tools such as clinical evaluations and neuroimaging provide valuable insights into individualized patient risk stratification; however these insights are often incomplete (5). This limitation has prompted researchers to identify reliable biomarkers with which to improve the accuracy of outcome prediction in patients with TBI.

Serum biomarkers such as glucose and potassium have garnered considerable attention in critical care research owing to their involvement in cerebral metabolism and homeostasis. The dysregulation of glucose metabolism is a well-established predictor of poor outcomes in TBI, given its prominent role in cerebral energy metabolism and inflammatory responses (6, 7). Similarly, as potassium homeostasis is critical for maintaining cellular membrane potential and neuromuscular function, its dysregulation is known to contribute to secondary brain injuries (8, 9). The glucose-to-potassium ratio (GPR) has recently been proposed as an indicator of metabolic stress in critically ill patients (10). however, despite the potential utility of GPR in MM-TBI, its specific prognostic value remains underexplored, with existing studies yielding inconsistent results (11).

Current research lacks a multifactorial approach combining GPR with established clinical assessment tools. One such tool is the Rotterdam score, which is based on neuroimaging findings and is widely used to assess TBI severity and predict potential outcomes (12). Few studies, however, have investigated the potential synergistic effect between the Rotterdam score and metabolic biomarkers such as GPR to enhance prognostic accuracy in patients with MM-TBI (13). This combined approach could provide a more comprehensive assessment of patient prognosis, consequently improving the clinical decision-making process.

## Methods and materials

### Study design and population

**Figure 1:**
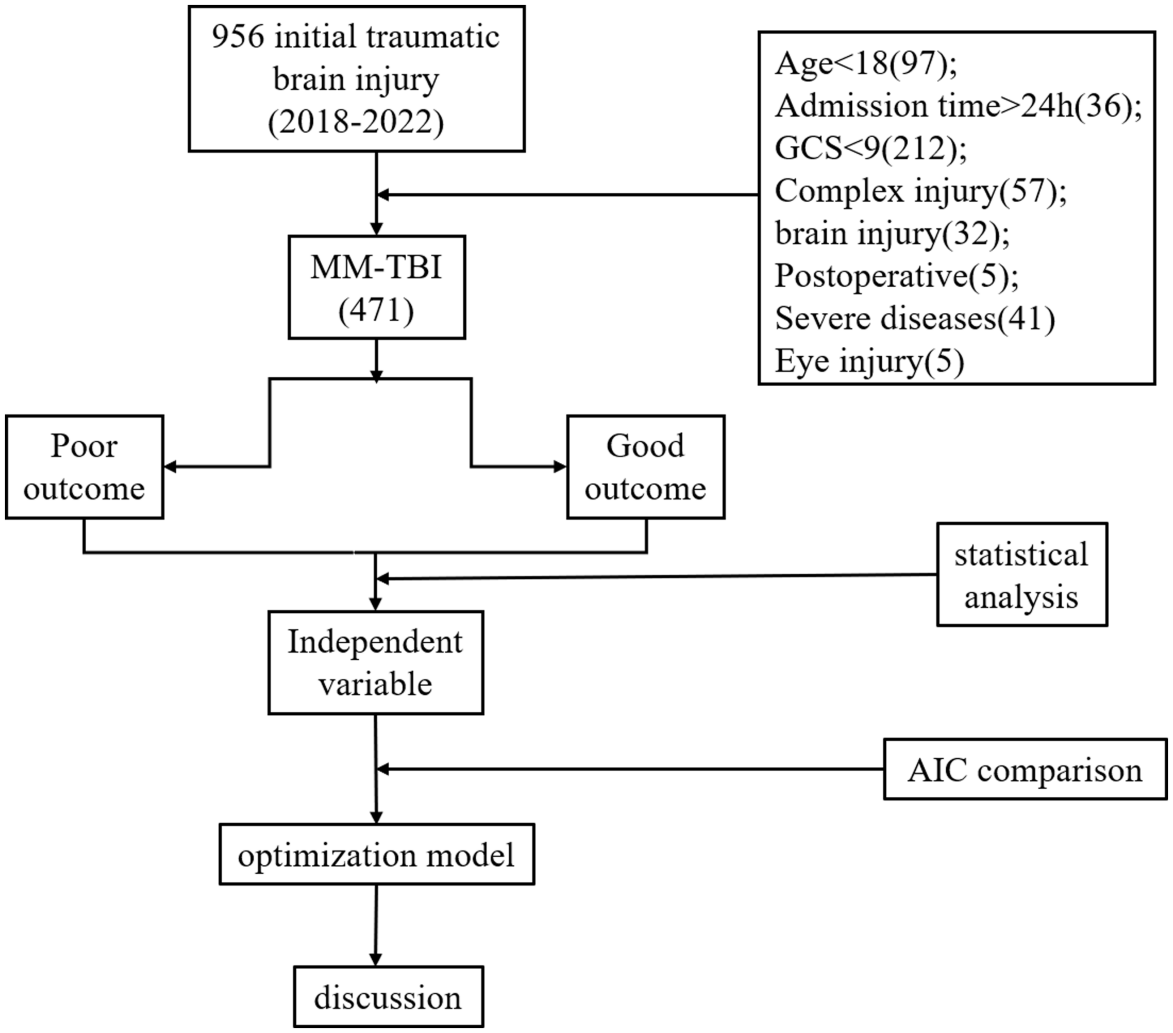
Flow diagram. The flow diagram, showing the data collection process.

This retrospective cohort study included patients admitted to the Second Affiliated Hospital of Fujian Medical University with MM-TBI between January 1, 2018 and December 31, 2022 (Figure 1). The inclusion criteria were as follows: (1) age ≥ 18 years; (2) MM-TBI diagnosed with a GCS score of 9–15 upon admission; and (3) availability of serum glucose and potassium levels at the time of admission. Patients with incomplete data, serious pre-existing neurological disorders, or those who required immediate surgical intervention were excluded. This study was approved by the Ethics Committee of the Second Affiliated Hospital of Fujian Medical University (2023, Review no. 287) and performed in accordance with the Declaration of Helsinki, revised in 2008. The need for informed consent was waived due to the retrospective nature of the study.

### Data collection

Clinical data including demographic information, medical history, and injury characteristics were obtained from electronic patient medical records. Key variables collected included the GCS score upon admission, Rotterdam score derived from computed tomography (CT) scans, and serum glucose and potassium levels. Patient prognoses were assessed using the modified Rankin Scale (mRS) at the time of discharge and 90 days later. Those who died or were left with severe disability (mRS ≥ 3) were categorized as having a poor prognosis, whereas those who recovered or had less than a moderate disability (mRS < 3) were categorized as having a good prognosis. GPR was calculated as the primary exposure variable, and patients were categorized into four groups based on quartiles to facilitate trend analysis. Propensity score matching (PSM) was used to mitigate selection bias within the cohort by matching patients based on baseline characteristics such as age, sex, and injury severity.

### Statistical analysis

Statistical analyses were conducted using R software (version 4.2.1). Continuous variables are presented as mean ± standard deviation (SD) or median and interquartile range (IQR), depending on their distribution, and were compared using the Student’s *t*- or Mann–Whitney *U* test, as appropriate. Categorical variables are expressed as frequencies and percentages and compared using the chi-squared or Fisher’s exact test, as appropriate. Univariate logistic regression was performed to identify risk factors for poor prognosis, and variables with a *p*-value < 0.10 were included in a multivariate logistic regression model to determine factors that were independent predictors of prognosis.

Restricted cubic spline (RCS) functions were utilized to explore potential nonlinear relationships between GPR and patient outcomes, adjusting for confounding variables such as age, sex, and Rotterdam score. The receiver operating characteristic (ROC) curve was used to evaluate the predictive efficacy of GPR independently and in combination with the Rotterdam score. The area under the curve (AUC) was utilized to assess model discrimination. Kaplan–Meier (K-M) survival curves were constructed to evaluate the association between GPR categories and patient outcomes, with differences assessed using the log-rank test. Statistical significance was set at *p* < 0.05 for all analyses.

## Results

### Baseline characteristics

Table 1 shows the baseline characteristics of the 471 included patients, 301 of whom were male (63.9%) and 170 female (36.1%). The median age was 55 (range, 18–95) years. Among the patients, 93 (19.8%) had hypertension and 40 (8.5%) had diabetes. A total of 48 patients (10.2%) experienced unfavorable outcomes, based on mRS scores of 3–6. There were no statistically significant differences between the favorable and unfavorable prognosis groups in terms of age, sex, body temperature, or systolic or diastolic blood pressure (*p* > 0.05); however, the Rotterdam score upon admission was significantly higher in the unfavorable than in the favorable prognosis group (*p* < 0.001). Additionally, serum analysis revealed that patients in the unfavorable prognosis group had a significantly higher GPR than those in the favorable prognosis group (3.38 vs. 2.00, respectively; *p* = 0.001). Significant differences were also observed in the white blood cell (WBC; *p* = 0.009) and neutrophil (*p* = 0.016) counts between the two groups.

**Table 1:** Demography of the study population.

Variable names	Overall	Unfavorable prognosis	Favorable prognosis	*p*-value
*N*	471			
Gender (%)				0.563
Male	170 (36.09)	33 (68.75)	268 (63.36)	
Female	301 (63.91)	15 (31.25)	155 (36.64)	
Age	55 (18–95)	53.5 (22–88)	55 (18–95)	
Hypertension (%)				0.993
Yes	93 (19.75)	10 (20.83)	83 (19.62)	
No	378 (80.25)	38 (79.17)	340 (80.38)	
Diabetes (%)				0.753
Yes	40 (8.49%)	3 (6.25)	37 (8.75)	
No	431 (91.51%)	45 (93.75)	386 (91.25)	
T	36.6 (36–38.8)	36.6 (36.2–37.8)	36.6 (36–38.8)	0.120
R	20 (12–30)	20 (12–27)	20 (14–30)	0.959
HR	80 (38–164)	80 (51–122)	80 (38–164)	0.275
SBP	132 (58–228)	131.5 (58–203)	133 (93–228)	0.670
DBP	80 (12–145)	79.5 (34–106)	80 (12–145)	0.724
Time to onset	4 (0.5–24)	4 (1–24)	4 (0.5–24)	0.471
Admission Rotterdam	2 (1–6)	3 (1–6)	2 (1–5)	<0.001
WBC	12.78 (4.12–36.97)	14.9 (5.71–35.4)	12.5 (4.12–36.97)	0.009
Neutrophil	10.33 (0.2–31.7)	12.675 (2.71–31.7)	10.2 (0.2–29.36)	0.016
Lymphocyte	1.46 (0.01–9.74)	1.64 (0.39–6.4)	1.41 (0.01–9.74)	0.379
Monocyte	0.7 (0.06–3.76)	0.745 (0.06–1.96)	0.7 (0.15–3.76)	0.166
RBC	4.46 (0.01–6.72)	4.43 (3.42–5.37)	4.48 (0.01–6.72)	0.904
Hemoglobin	134 (0.01–180)	137 (107–166)	134 (0.01–180)	0.309
Blood platelet	223 (14.7–470)	228.5 (120–329)	223 (14.7–470)	0.806
Urea_nitrogen	5.02 (1.03–80.5)	4.68 (2.67–9.86)	5.11 (1.03–80.5)	0.468
Creatinine	64.6 (0.06–486)	64.3 (38.4–110.6)	64.6 (0.06–486)	0.628
Uric acid	0.08 (0.03–369)	356.5 (128–566)	335 (5.34–867)	0.663
GPR	336 (5.34–867)	2.38 (1.355–5.867)	2 (0.87–8.08)	0.001

T, Temperature; R, Respiratory; HR, Heart rate; SBP, Systolic blood pressure; DBP, Diastolic blood pressure; WBC, White blood cell; RBC, Red blood cell; GPR, Glucose potassium ratio.

### Independent risk factors

Univariate logistic regression analysis identified the Rotterdam score upon admission, GPR, WBC, and neutrophil count as potential risk factors for poor prognosis. These factors were subsequently included in a multivariate logistic regression model (Table 2), which revealed that the Rotterdam score upon admission and GPR were independent predictors of poor outcomes (*p* < 0.001 and *p* = 0.002, respectively).

**Table 2:** The results of multivariate logistic regression analysis.

Variable name	Z_value	*p*-value	OR	Lower_OR	Upper_OR
Admission_Rotterdam	5.676	<0.0001	2.717	1.949	3.905
Neutrophil	1.038	0.300	1.1360	0.900	1.466
WBC	−0.724	0.469	0.918	0.720	1.500
GPR	3.082	0.002	1.663	1.190	2.300

WBC, White blood cell; GPR, Glucose potassium ratio.

### Association between GPR and clinical outcomes

GPR (median, 2.03; range, 0.87–8.08) was identified as an independent risk factor for poor prognosis in patients with MM-TBI. The odds ratio (OR) was 1.663, indicating that for each unit increase in GPR, the risk of unfavorable outcomes increased by 66.3%. A trend analysis was conducted after grouping GPR into quartiles (Q1, 0.87–1.69; Q2, 1.70–2.02; Q3, 2.03–2.43; Q4, 2.44–8.08), with Q1 serving as the reference. The results showed a progressive increase in the risk of unfavorable outcomes with higher GPR levels (OR_trend_ = 2.79, *P*_trend_ < 0.001). Compared to Q1, the OR for Q3 was 3.17 (*p* = 0.05), and that for Q4 was 6.35 (*p* < 0.001), indicating a substantially higher risk in Q4 compared to Q1 (Table 3).

**Table 3:** GPR trend test.

GPR	Outcome	
	OR (95% CI)	*p*-value
Q1	ref	ref
Q2	2.59 (0.79,8.52)	0.12
Q3	3.17 (0.99,10.14)	0.05
Q4	6.35 (2.11,19.07)	<0.001
*P* for trend	2.79 (1.63,4.78)	<0.001

Q1, 0.87–1.69; Q2, 1.70–2.02; Q3, 2.03–2.43; Q4, 2.44–8.08. OR, odd ratio; CI, confidence interval.

### Nonlinear relationship between GPR and prognosis

**Figure 2:**
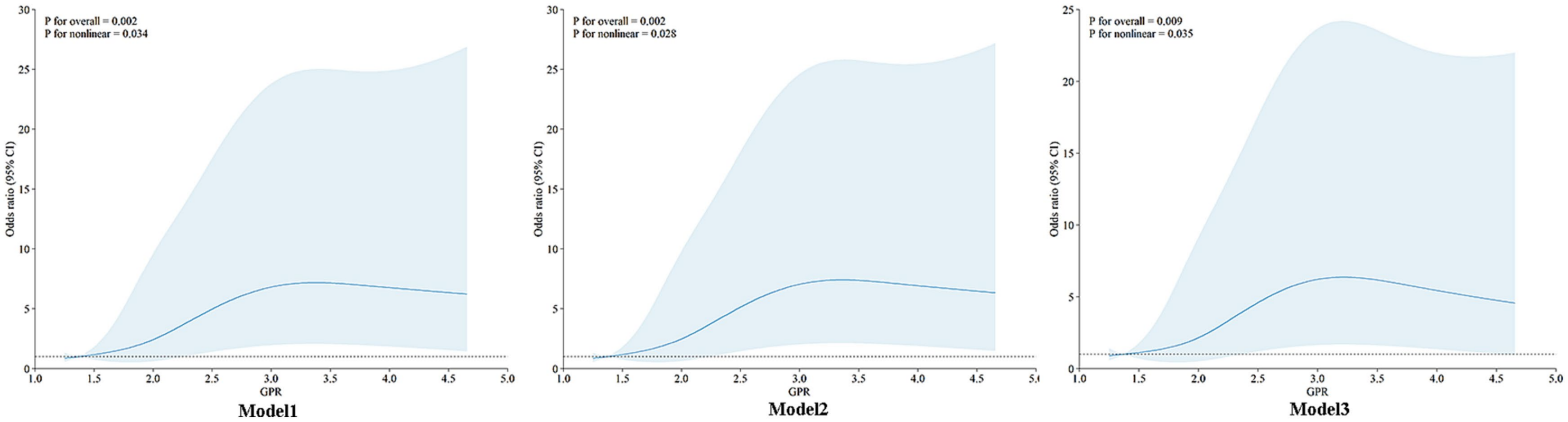
Restricted cubic spline. Three distinct adjustment levels were used in a restricted cubic spline (RCS) model. A nonlinear connection was shown by the unadjusted model (Model 1) (*P* for nonlinearity = 0.034). A nonlinear link was also seen in the partially corrected model (Model 2), which took gender and age into account (*P* for nonlinearity = 0.028). This correlation was maintained in the completely adjusted model (Model 3) (*P* for nonlinearity = 0.035). The findings showed a consistent nonlinear correlation between prognosis and GPR.

An RCS model with three different adjustment levels was utilized to evaluate the nonlinear relationship between GPR and prognosis, as shown in Figure 2. The unadjusted model (model 1) demonstrated a nonlinear association (*P*_nonlinearity_ = 0.034). This nonlinear association was also maintained in the partially adjusted model, which accounted for age and sex (model 2; *P*_nonlinearity_ = 0.028), as well as the fully adjusted model (model 3; *P*_nonlinearity_ = 0.035). The results indicated a stable nonlinear relationship between GPR and prognosis. Further analysis using multiple regression equations revealed that in model 1, each unit increase in GPR was associated with a 55.9% increase in the risk of unfavorable outcomes (OR = 1.559; 95% confidence interval: 1.176–2.068; *p* = 0.002). This risk remained consistent even after partial (model 2: OR = 1.559; *p* = 0.002) and full (model 3: OR = 1.435; *p* = 0.020) adjustment.

### Propensity score matching

**Figure 3:**
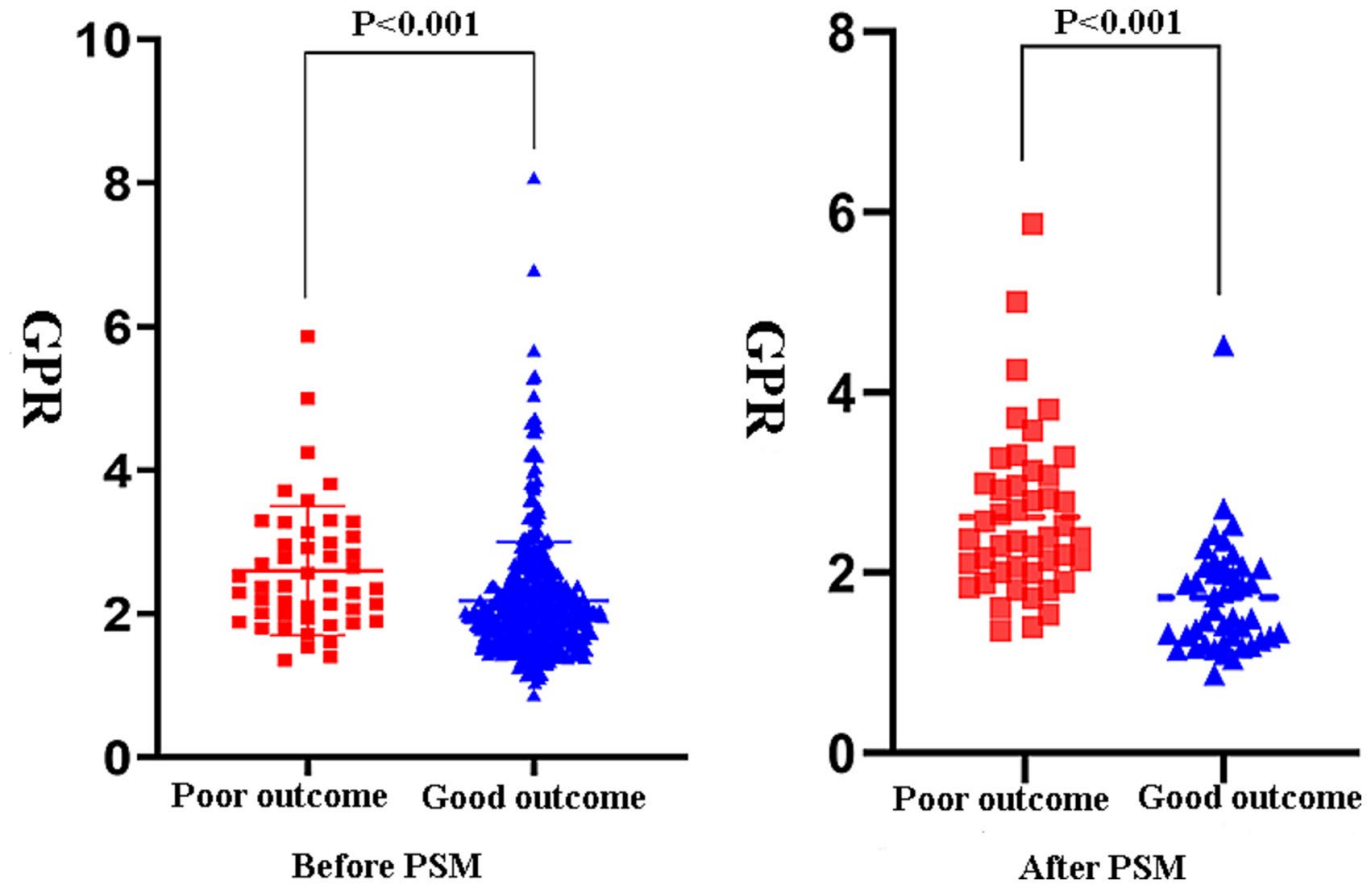
The favorable and poor prognosis groups’ GPRs still differed statistically significantly after matching (*p* = 0.004). Figure 3 shows the differences between the two groups before and after PSM, showing that GPR was substantially different before and after matching (*p* < 0.001).

Given the retrospective nature of this study, propensity score matching (PSM) was utilized to mitigate selection bias. After matching, there was still a statistically significant difference in GPR between the favorable and unfavorable prognosis groups (*p* = 0.004) (Table 4). The differences before and after PSM are depicted in Figure 3, which shows that GPR remained significantly different between the two groups before and after PSM (*p* < 0.001).

**Table 4:** The characteristics of variable after PSM.

Variable names	Overall	Favorable prognosis	Unfavorable prognosis	*p*-value
*n*	84	42	42	
Age	52.5 (18–88)	52 (18–69)	53 (22–88)	0.466
Gender (%)				1.000
Female	24 (28.57)	12 (28.57)	12 (28.57)	
Male	60 (71.43)	30 (71.43)	30 (71.43)	
Hypertension (%)				1.000
No	69 (82.14)	35 (83.33)	34 (80.95)	
Yes	15 (17.86)	7 (16.67)	8 (19.05)	
Diabetes (%)				1.000
No	79 (94.05)	40 (95.24)	39 (92.86)	
Yes	5 (5.95)	2 (4.76)	3 (7.14)	
SBP	131 (58–228)	128 (93–228)	132 (58–203)	0.986
DBP	80 (34–125)	80 (53–125)	79.5 (34–106)	0.827
Time_to_onset	4 (1–24)	4 (2–24)	4 (1–24)	0.937
Admission_Rotterdam	3 (1–6)	3 (1–5)	3 (1–6)	0.923
WBC	14.695 (5.71–35.4)	14.4 (7–30.78)	14.695 (5.71–35.4)	0.831
Neutrophil	12.215 (2.71–31.7)	10.7 (4.7–27.53)	12.915 (2.71–31.7)	0.680
Lymphocyte	1.58 (0.4–9.74)	1.5 (0.4–9.74)	1.63 (0.4–5.1)	0.435
Monocyte	0.8 (0.3–2.3)	0.8 (0.3–2.3)	0.775 (0.44–1.96)	0.994
RBC	4.475 (3.42–5.37)	4.56 (3.44–5.32)	4.43 (3.42–5.37)	0.471
Hemoglobin	137.5 (81–166)	137.5 (81–158)	137.5 (107–166)	0.901
Blood_platelet	232 (120–336)	237.5 (121–336)	228.5 (120–329)	0.453
Urea_nitrogen	4.755 (1.84–10.55)	4.87 (1.84–10.55)	4.68 (2.67–9.86)	0.728
Creatinine	64.4 (38.4–110.6)	64.4 (39.4–99)	64.3 (38.4–110.6)	0.897
Uric_acid	343 (117–566)	327 (117–530)	356.5 (128–566)	0.306
GPR	2.167 (1.355–5.867)	1.999 (1.403–4.526)	2.456 (1.355–5.867)	0.004

SBP, Systolic blood pressure; DBP, Diastolic blood pressure; WBC, White blood cell; RBC, Red blood cell; GPR, Glucose potassium ratio; PSM, Propensity score matching.

### Predictive value of GPR for unfavorable outcomes

**Figure 4:**
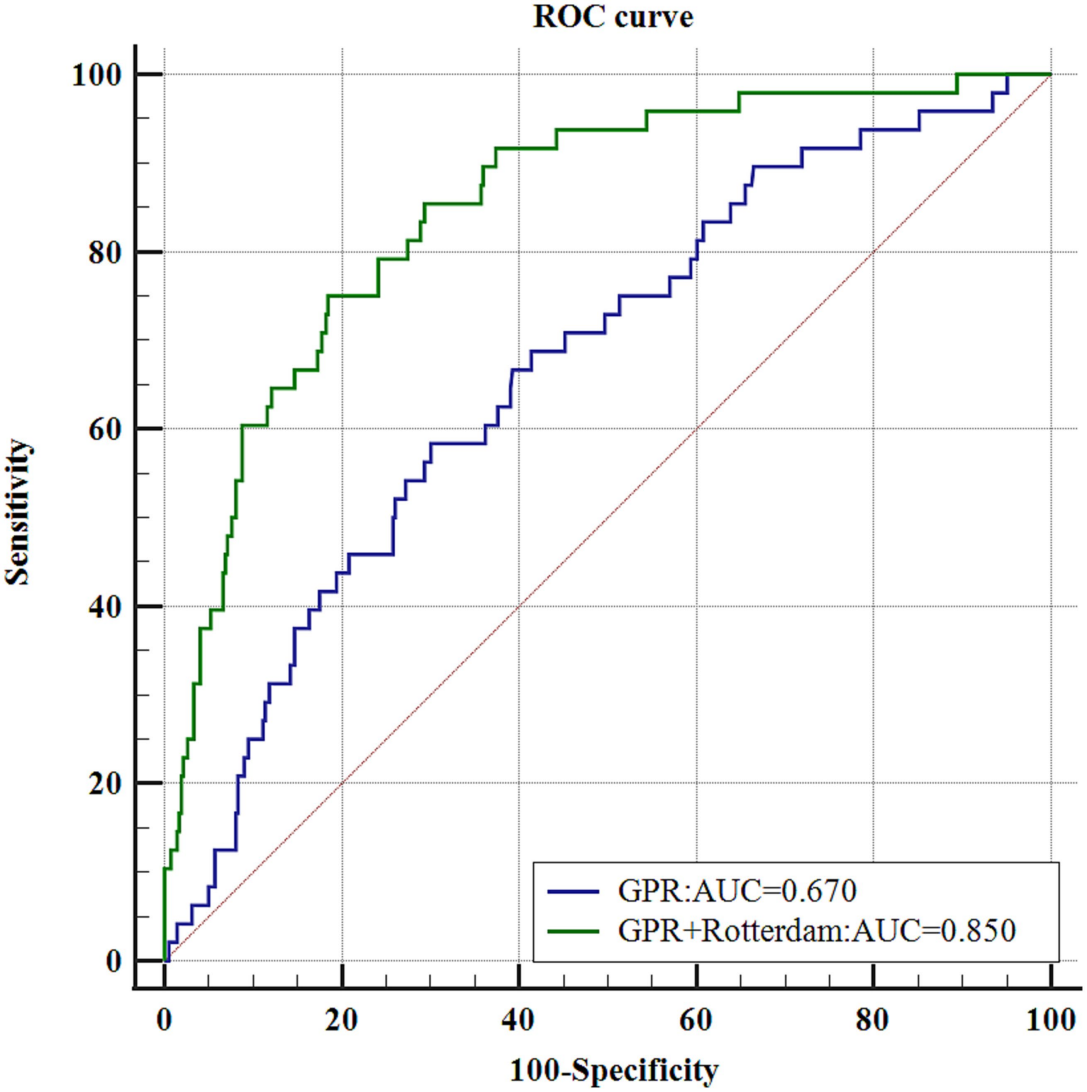
GPR and GPR + Rotterdam ROC curve. Showed an area under the curve (AUC) of 0.670 (*p* < 0.0001). Combining GPR with the admission Rotterdam score significantly improved the predictive performance, resulting in an AUC of 0.850 (*p* < 0.0001).

**Figure 5:**
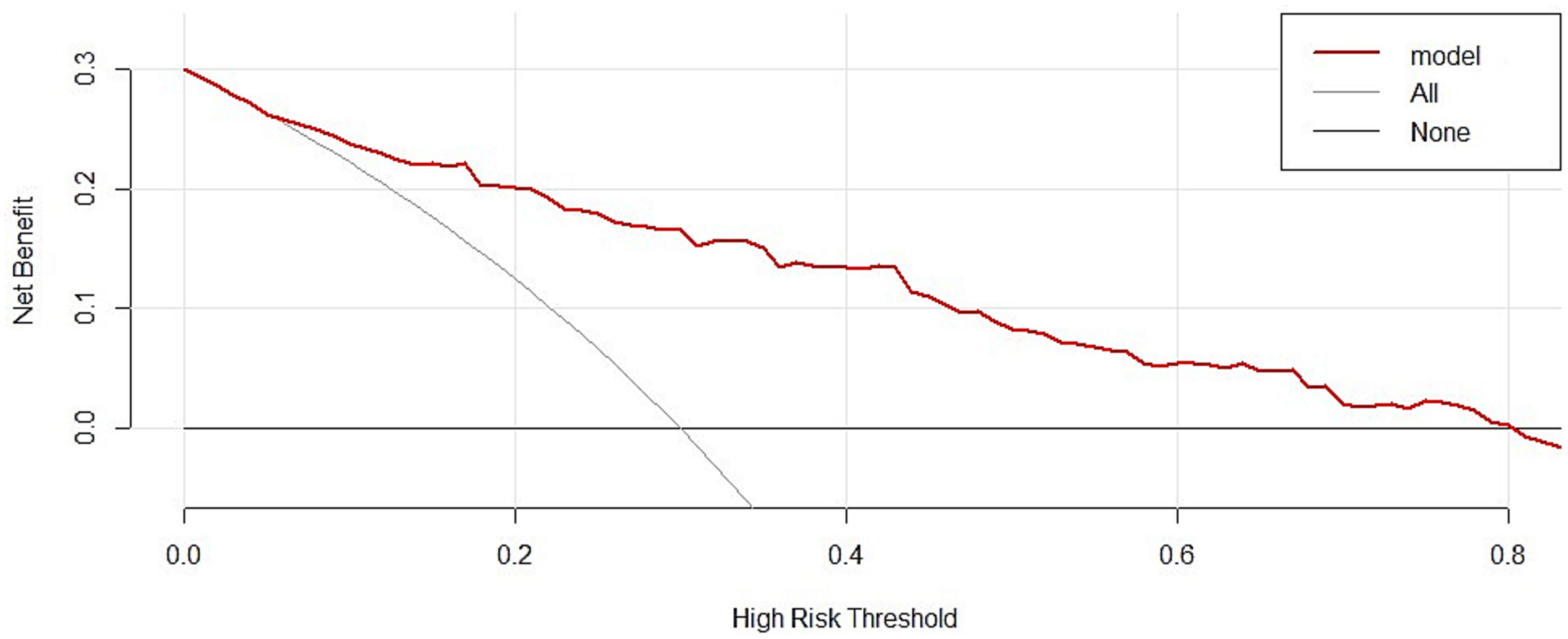
GPR DCA curve. Showed the clinical decision curve also demonstrated that combining GPR with imaging assessments could benefit approximately 80% of patients.

The utility of GPR as an independent risk factor in predicting unfavorable outcomes was evaluated using an ROC curve. Figure 4 shows an AUC of 0.670 (*p* < 0.0001) with a sensitivity of 58.33%, specificity of 69.98%, Youden index of 0.283, and a cut-off value for GPR of > 2.286. Combining GPR with the Rotterdam score upon admission significantly improved its predictive performance, resulting in an AUC of 0.850 (*p* < 0.0001) with a sensitivity of 75.00% and specificity of 81.56%, indicating a 28% improvement in predictive accuracy. Additionally, the clinical decision curve demonstrated that combining GPR with imaging assessments could improve prognostic accuracy in approximately 80% of patients (Figure 5).

**Figure 6:**
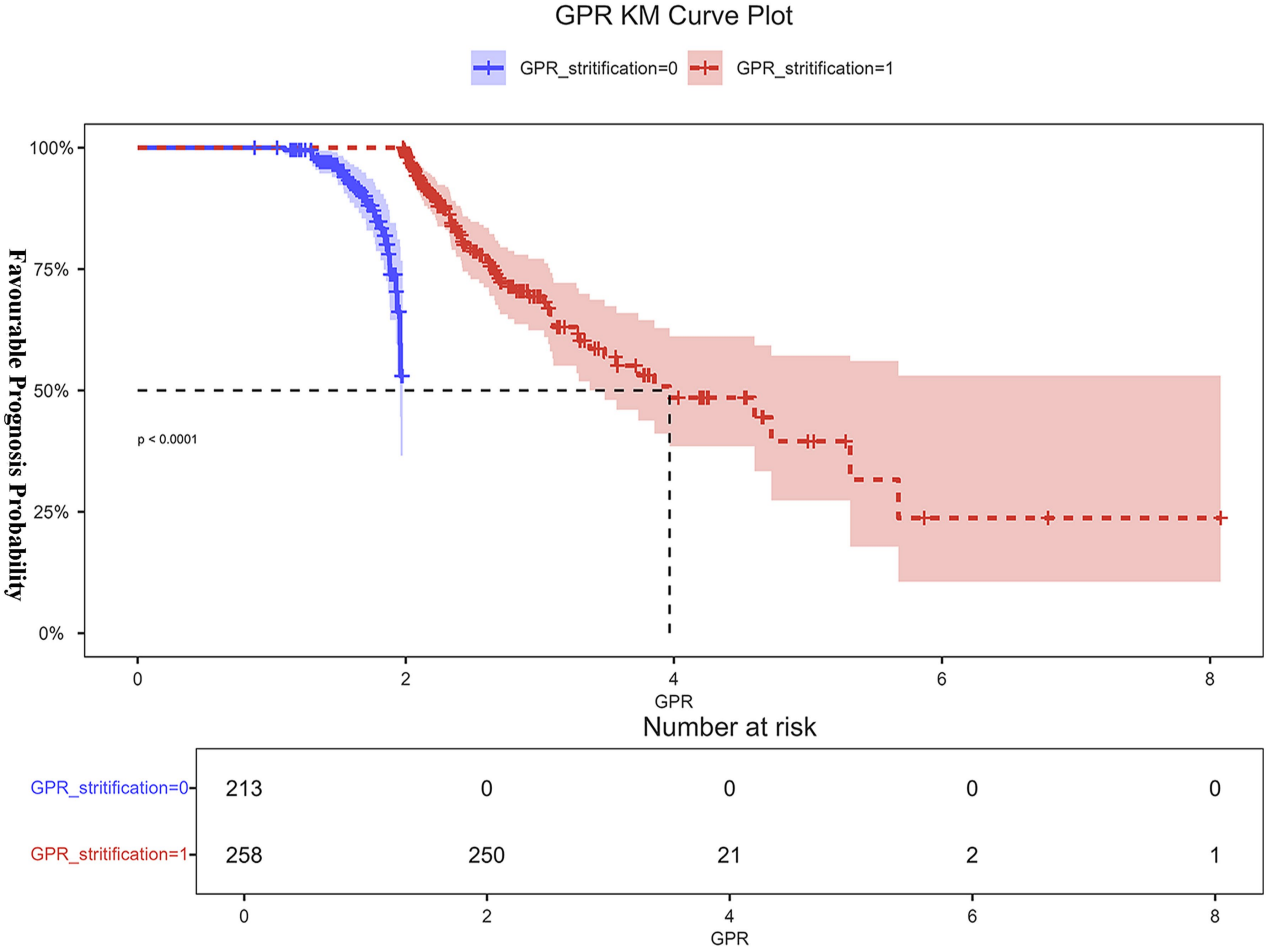
GPR K-M plot. When GPR was dichotomized at the threshold value (>2.286), the Kaplan–Meier (K-M) curve showed that GPR was a significant predictor of 90-day outcomes in patients with MM-TBI (*p* < 0.0001). At 90 days after follow-up, the positive prognosis percentage was more than 50% when the GPR was less than 2.286. The rate of positive prognosis, however, decreased linearly when GPR rose over 2.286 and stabilized when GPR surpassed 6.

The K–M curve, generated by dichotomizing GPR at the cut-off value (>2.286), indicated that GPR was a significant predictor of 90-day outcomes in patients with MM-TBI (*p* < 0.0001). When GPR was <2.286, the favorable prognosis rate was >50% at 90 days post-discharge; however, as GPR increased beyond 2.286, the rate of favorable prognoses declined linearly and stabilized when GPR was >6 (Figure 6).

## Discussion

TBI triggers a complex cascade of biochemical and physiological responses that contribute to both primary and secondary injury mechanisms (2). Secondary injury involves metabolic dysregulation, neuroinflammation, and immune responses, all of which have a considerable effect on patient outcomes (3). Recent studies have emphasized the role of metabolic biomarkers such as glucose and potassium in determining the severity of these responses (14). GPR, a novel biomarker, encapsulates both metabolic and immunological imbalances, allowing for a more nuanced prediction of MM-TBI prognosis (10). The prognostic significance of glucose and potassium levels has been extensively investigated in various critical care settings, including TBI (11). Hyperglycemia commonly occurs post-TBI and is primarily driven by the stress-induced release of catecholamine and activation of the hypothalamic–pituitary–adrenal axis. Persistent hyperglycemia is associated with increased oxidative stress, exacerbation of neuronal damage, and poor clinical outcomes. Similarly, abnormal potassium levels, particularly hypokalemia, can indicate a disruption in cellular homeostasis which may lead to neuronal hyperexcitability and the subsequent exacerbation of secondary brain injury (6, 15).

Although numerous studies have examined individual metabolic biomarkers, relatively few have systematically investigated the combined influence of glucose and potassium imbalance on TBI outcomes (2, 6, 16). Although Huang et al. (17) posited that GPR was an independent risk factor affecting the prognosis of craniocerebral injury, they did not distinguish the severity of the disease among patients with craniocerebral injury. In a description of the associated risk factors after surgery for severe head injury, it was mentioned that GPR, as an independent risk factor, has a unique predictive value for prognosis, although the predictive effect on the prognosis of patients with MM-TBI at the time of admission was not addressed (18). Furthermore, a study by Marini et al. (19) focused primarily on the prognostic role of GPR in general or mild-to-severe TBI. Current literature, however, lacks large-scale studies specifically investigating GPR in the context of MM-TBI, creating a gap in our understanding of its role in clinical decision-making. The findings from our study demonstrated that higher GPR values were significantly associated with an increased risk of unfavorable outcomes, thus supporting their prognostic value in patients with MM-TBI.

The mechanism by which the GPR predicts MM-TBI prognosis likely involves an interplay between metabolic dysregulation, inflammation, and the immune response. TBI triggers an acute inflammatory response that is characterized by the release of pro-inflammatory cytokines (such as interleukin-1β, interleukin-6, and tumor necrosis factor alpha) as well as the activation of microglia and astrocytes (20). Hyperglycemia exacerbates the inflammatory cascade by promoting oxidative stress and the formation of advanced glycation end products, which further enhance cytokine production and perpetuate neuronal injury (7). Potassium plays a critical role in maintaining cellular ionic balance and regulating immune cell function. Hypokalemia impairs T cell function and modulates the activity of immune cells such as neutrophils and macrophages, which are crucial during the acute immune response to brain injury (21). Therefore, disrupted potassium homeostasis can lead to heightened inflammatory activity, further aggravating secondary injury mechanisms in patients with TBI.

GPR as a biomarker, therefore, may reflect the balance between two key processes: the glucose-induced exacerbation of inflammation and the role of potassium in modulating the immune response (22). A higher GPR may indicate an elevated inflammatory state, contributing to poor clinical outcomes. Conversely, a lower GPR may suggest better metabolic control and a more regulated immune response, correlating with an improved prognosis. These findings are in agreement with our results, which showed a stable nonlinear relationship between GPR and prognosis using RCS models, further underscoring the significance of GPR as a prognostic biomarker in MM-TBI.

Despite the potential utility of GPR as a prognostic marker in MM-TBI, this study has limitations. First, its retrospective design introduces selection bias and limits the generalizability of the findings. Additionally, the exact threshold for clinically relevant GPR levels remains uncertain, with varying cut-off values reported for prognostic significance. A mechanistic understanding of how the GPR influences long-term outcomes, particularly its impact on the immune system, is still evolving and requires further investigation.

### Advantages and limitations

This study is among the first to systematically evaluate the prognostic value of GPR in MM-TBI, contributing to a growing body of evidence on the relationship between metabolic biomarkers with TBI outcomes. A large sample size enhanced the statistical power in this study, while multivariate logistic regression and RCS models provided insights into the nonlinear relationship between GPR and prognosis. Integrating GPR with clinical tools such as the Rotterdam score showed potential for improving individualized risk stratification and patient management.

This study had some limitations that warrant discussion. First, all data were collected from a single institution, resulting in a relatively homogeneous patient population with limited demographic diversity. This lack of diversity inherently limits the external validity of our results, meaning the findings may not be directly applicable to other hospitals, regions, or patient groups. Furthermore, despite the use of PSM to mitigate selection bias, it cannot address unmeasured confounders or the inherent narrow scope of a single-center dataset, meaning residual selection bias was possible. Second, the optimal threshold for clinically significant GPR remains unclear. The variability in the reported cut-off values among relevant studies complicates the definition of the appropriate GPR level for prognostic purposes. Third, this study primarily assessed short-term outcomes (90-day prognosis), meaning the long-term implications of GPR in patients with MM-TBI were not assessed. This lack of long-term follow-up data restricts our understanding of the effects of GPR on chronic MM-TBI outcomes and recovery. Finally, the mechanistic pathways through which GPR affects the immune and inflammatory responses in MM-TBI remain unclear, thus limiting the clinical applicability of GPR as a predictive biomarker and necessitating further experimental studies.

Future studies should focus on prospective multi-center studies to validate our results across a broader spectrum of patients and settings. Including multiple study locations and a more diverse patient demographic could validate the prognostic utility of GPR and explore its long-term effects in patients with MM-TBI, given its potential to reflect metabolic, inflammatory, and immune activity that may influence chronic outcomes in patients with MM-TBI. Additionally, mechanistic studies involving animal models are needed to elucidate the specific pathways through which GPR influences the immune and inflammatory responses. Such insights could pave the way for targeted interventions aimed at modulating these pathways, ultimately improving patient outcomes.

## Conclusion

GPR is a viable and readily available biomarker for predicting the prognosis of patients with MM-TBI. Furthermore, the Rotterdam score upon admission can serve as an important complement to GPR, enhancing its ability to predict the prognosis of patients with MM-TBI and facilitating clinicians’ assessments of likely prognostic progression, guiding the therapeutic decision-making process.

## Data Availability

The raw data supporting the conclusions of this article will be made available by the authors, without undue reservation.
